# Understanding of Excellent Mechanical Performance of 304L Manufactured by Optimal Selective Laser Melting (SLM) Conditions

**DOI:** 10.3390/ma16041661

**Published:** 2023-02-16

**Authors:** Yaxin Ma, Yifei Gao, Lei Zhao, Hong Zhang, Dongling Li, Lixia Yang, Chuntang Yu

**Affiliations:** 1 Central Iron & Steel Research Institute, NCS Testing Technology Co., Ltd., Beijing 100081, China; 2 Chengdu Aeronautic Polytechnic, Chengdu 610100, China; 3 Failure Mechanics and Engineering Disaster Prevention and Mitigation Key Laboratory of Sichuan Province, College of Architecture and Environment, Sichuan University, Chengdu 610065, China; 4 School of Materials Science and Engineering, Chongqing University of Technology, Chongqing 401320, China

**Keywords:** selective laser melting (SLM), 304 stainless steel, microstructure, mechanical properties

## Abstract

The optimal SLM conditions of 304L stainless steel were obtained by single factor and orthogonal tests. Results indicated that the optimal hardness (75 HRB) and Relative Density (RD 99.24%) could be obtained when the laser output power was 190 W, the scanning distance was 0.09 mm and the scanning speed was 800 mm/s. The microstructure of fish scales was uniform and compact with a few pores in the optimal sample. The fine particles were randomly distributed near the edge of the molten pool, and some preferred granular columnar crystal structures were formed. Abundant entanglement dislocations were observed between cell structures, forming dislocation clusters. Spherical nano-precipitates, rich in Si, Mn, and O, were also observed near cell structures. The mechanical properties of the specimens were highly anisotropic, and there were obvious necking and ductility at the tensile fracture.

## 1. Introduction

Recently, additive manufacturing (AM) has attracted wide attention due to its ability to produce complex components without molding while maintaining structural strength, significantly improving production efficiency and reducing costs [1,2,3,4,5]. Among the various AM techniques, selective laser melting (SLM) is the most prominent and efficient method [2,4]. It can print different features with fine microstructures. Due to ultrafast cooling, smooth and shiny surfaces can be achieved with high mechanical strength. This technique has been well applied in metal alloys, such as Inconel 718, 316L, Ti64, high-entropy alloys, etc. [6,7,8,9,10,11]. 

304L stainless steel (SS) is a crucial metallic material for industrial applications, such as marine environments, chemical plants, and especially nuclear power plants, due to its great mechanical performance and excellent anticorrosion properties [6,7]. However, there are several papers about 304L manufactured by SLM. Guan et al. [4,8,12] studied the influence of the powder layer thickness, construction direction, component overlap, volume energy density, and cap angle on the mechanical properties, and obtained excellent strength and ductility by optimizing the SLM parameters for 304L SS components. Hou et al. [13] studied the microstructures, tensile properties, and mechanical anisotropy of 304L SS parts processed by SLM. Under the optimized laser processing parameters, fine austenite particles and nano cellular structures with a grain dimension of about 500 nm were obtained. Jeong et al. [14] investigated the effect of the metastable δ ferrite and twin-induced plasticity on the strain-hardening behavior of 304L austenitic SS processed by SLM. It was found that the ultrafine δ ferrite maintained coherence with the γ austenite matrix in an un-deformed state, which interacted with dislocations during the plastic deformation. Lee et al. [15] investigated the high-pressure torsion (HPT) induced significant strengthening of the 304L SS during SLM by nano-mechanical analysis and microstructural characterization. The results showed that the SLM 304L SS achieved significant HPT-induced strengthening, resulting from the synergy effect of dislocation recombination, grain refinement, and martensitic transformation, where the FCC(γ) transformed into HCP(ε) and BCC(α) martensite [16,17,18,19]. 

On the one hand, there are few and insufficient research studies on 304L process parameters, microstructures and mechanical properties. On the other hand, unavoidable defects and the difficulty in controlling the structure are crucial conditions limiting the advancement of SLM technology [2,3,20]. Meanwhile, different imperfections can be produced due to variations in equipment, manufacturing conditions, and situations [2,3,4,21]. Therefore, optimizing the process conditions, reducing defects, and obtaining specimens with excellent mechanical properties during the forming process are important for SLM processes.

To obtain excellent mechanical properties of 304L SS components from SLM, further studies were carried on process parameter optimization and microstructure characterization. In the present study, single-factor and orthogonal experiments were performed to investigate the effects of laser energy, hatch space, and scan rate on the RD and hardness of the SLM components. The relative optimum processing parameters were achieved at the layer thickness of 0.03 mm. The microstructures and mechanical performance of the SLM samples were analyzed, which provided an optimal condition for the high-quality SLM processing of 304L SS. 

## 2. Materials and Experiment Details

### 2.1. Materials

**Figure 1:**
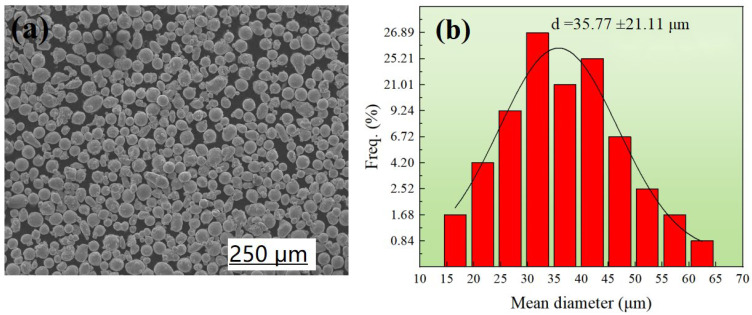
(**a**) SEM image of the particles in the 304L SS powder and (**b**) the normalized particle size distributions.

The 304L SS powder was prepared by vacuum air mist, and its typical chemical components are shown in Table 1. Figure 1a shows the spherical particulate morphology of powder; most of the particles had smooth surfaces. The particles had a relatively uniform size distribution with a mean diameter from 15 μm to 65 μm. The size distributions are shown in Figure 1b. The bulk density was 4.10 g/cm^3^, while the Hall flow rate was 18 s/50. The samples were as-built using a metal 3D printer (FS121M).

### 2.2. Experimental Details

**Figure 2:**
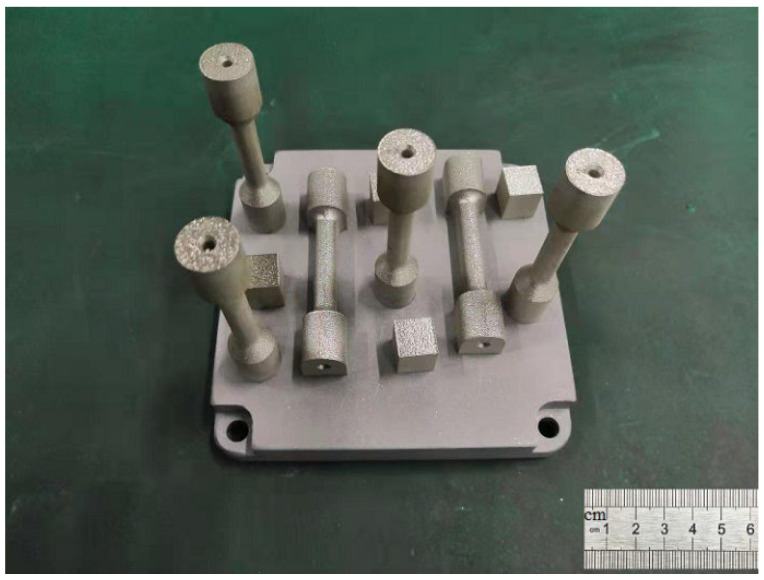
The metallographic and tensile samples.

The measurements were designed in three stages to obtain relatively optimal forming conditions. (1) Confirm the scope of the relatively optimal process conditions. The layer thickness of 0.03 mm, the laser power (170 W, 190 W, 200 W), the hatch space (0.06 mm, 0.09 mm, 0.12 mm), and the scanning speed (600 mm/s, 800 mm/s, 1000 mm/s) were adjusted individually, producing 9 groups of samples (the size was 12 mm × 12 mm × 12 mm). The scanning direction after each layer was rotated 67°. The density and hardness of the 304L sample after forming were characterized to establish the range of the measurement conditions. (2) Use the orthogonal analysis to determine the relative optimal process parameters. (3) Print the metallographic and tensile samples (as shown in Figure 2) under optimal conditions to establish their formed structures and the associated mechanical strength.

The SLM 304L specimens were etched with aqua regia. Microstructure and imperfections were characterized with a metallographic microscope (GX51) and a 3-D X-ray microscope (Skyscan2214, Bruker) with an emission current of 35 mA at a bias of 130 kV and a resolution of 3 μm. The density and hardness were measured with a direct-reading solid density meter (MH-600A) and a hardness tester (HR-150A, Rockwell). The mechanical properties were tested by a universal tensile testing machine (E45, MTS). Microstructure observation and fracture analysis were carried out using a scanning electron microscope (SU3500) and a field-emission transmission electron microscope (FETEM, Tescan G2 F20, FEI).

## 3. Results and Analysis

### 3.1. RD Analysis

**Figure 3:**
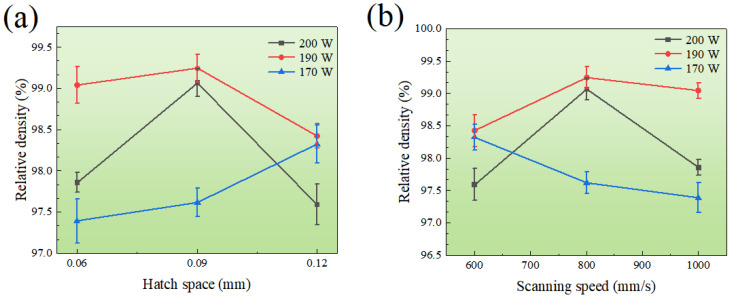
The RD depended on hatch space (**a**) and scanning speed (**b**) under different heating power levels of 170, 190 and 200 W.

Relative Density (hereinafter named the RD) is defined as the ratio between the actual measured density and theoretical density of the SLM 304L SS. The variations of RD with linear scan speed, hatch space and heating power of the laser beam are shown in Figure 3. As can be seen, the maximum RD reached 99.24%, which was higher than the reported literature value [8,12], while the minimum RD was 97.39%. When the heating laser power was 190 or 200 W, the RD increased first and then decreased with the increasing of the linear scanning rate or hatch space. However, as the laser power was reduced to 170 W, the RD value gradually decreased with the increasing of scanning speed and hatch space. The highest RD was obtained at the heating power of 190 W, the linear scan rate of 800 mm/s, and the hatch space of 0.09 mm. The RD varied greatly under a combination of different process parameters, which may be related to the porosity of the samples.

**Figure 4:**
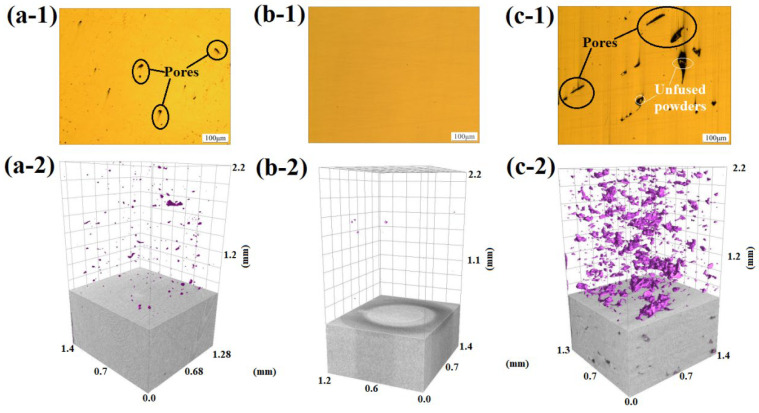
2-D and 3-D pore distribution plots of different printing parameters: (**a-1**,**a-2**) 200 W, 0.09 mm, 800 mm/s; (**b-1**,**b-2**) 190 W, 0.09 mm, 800 mm/s; and (**c-1**,**c-2**) 170 W, 0.09 mm, 800 mm/s.

In order to investigate the mechanism responsible for the difference in the RD, three specimens under different processing conditions were chosen for analyzing the pore size distribution using a local micro-CT. After 3-D reconstruction and analysis, the test results are plotted in Figure 4. Under the conditions of fixed layer thickness of 0.03 mm, hatch space of 0.09 mm, scanning speed of 800 mm/s and laser power of 170 W, the pores with a broad size distribution were formed. When the laser power was increased to 200 W, fewer large pores were formed with dominant small pores. At 190 W, the porosity was the least. Therefore, the distribution of the sample pore size, shape, position, and population is closely related to the laser power, which determines the RD. In addition, due to the limited resolution and other reasons, very small pores cannot be quantitatively analyzed, which may result in the porosity obtained in the experiment being less than the real value.

### 3.2. Hardness Characterization

**Figure 5:**
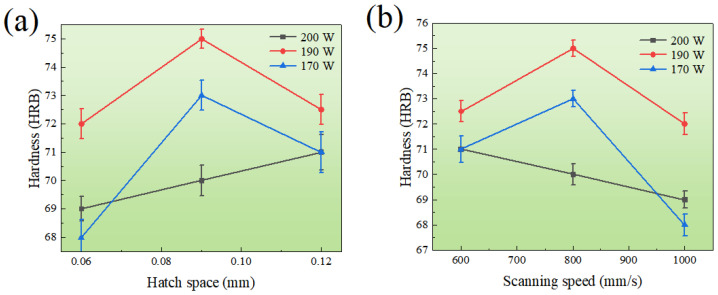
The hardness depended on hatch space (**a**) and scanning speed (**b**) under different heating power levels of 170, 190 and 200 W.

The hardness variation of the SLM 304L SS under different laser power, hatch space, and scanning speed is shown in Figure 5. The maximum hardness of the material was 75 HRB, while the minimum hardness was 68 HRB. Specifically, when the laser power was 190 or 200 W, the hardness initially improved and then reduced with increased scanning rate and hatch space, respectively. At the laser power of 170 W, the hardness decreased with increase of the scanning speed and decrease of the hatch space. The highest hardness was obtained at the heating power of 190 W, the linear scan rate of 800 mm/s, and the hatch space of 0.09 mm. 

### 3.3. Orthogonal Test and Range Analysis

The orthogonal tests and the range analysis were conducted to process the single-factor results. Table 2 lists the RD and the hardness from the orthogonal analysis, while the corresponding ranges are summarized in Table 3. The data indicated that the sensitivity of the RD complies with the following sequence: laser power > scan rate > hatch space. Meanwhile, the sensitivity of the hardness to the parameters follows the sequence of laser power > hatch space > scan rate. These results agreed well with the obtained single-factor experiment. Laser power is the most important parameter, while hatch spacing and scanning speed have little effect on RD and hardness. In addition to this, when the thickness of the layer was 0.03 mm, the optimal forming conditions were laser heating power of 190 W, scanning speed of 1000 mm/s and hatch space of 0.09 mm; the RD value achieved the highest value of 99.24% at the relatively high hardness of 75 HRB, which is better than results reported in the literature [8,12]. 

### 3.4. Microstructure and Micromechanical

**Figure 6:**
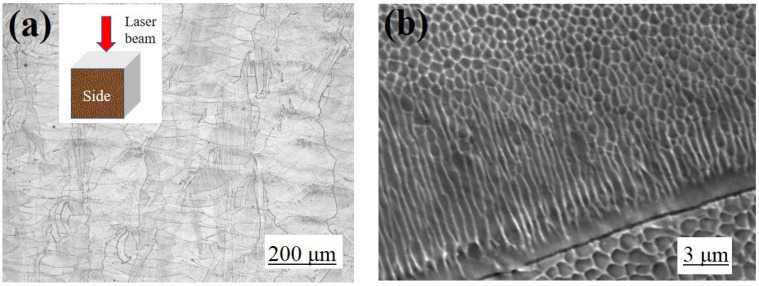
Microstructure: (**a**) side, (**b**) high magnification SEM image of (**a**).

Metallographic polishing and etching were carried out on the samples with the highest RD and hardness, and the corresponding microstructural morphologies are shown in Figure 6. The obvious “fish scale” texture without any holes and other defects from deposited molten pool are presented in Figure 6a. Additionally, the further magnified SEM image shown in Figure 6b revealed that the fine uniform columnar and cellular microstructure were formed during the rapid cooling after laser melting, instead of the traditional austenite structure. The uniform columnar and cellular structures were densely packed and grow along the thermal diffusion direction near the boundary of the molten pool, without distinctive metallurgical transition, which suggested that a good blending was achieved during the SLM deposition. 

**Figure 7:**
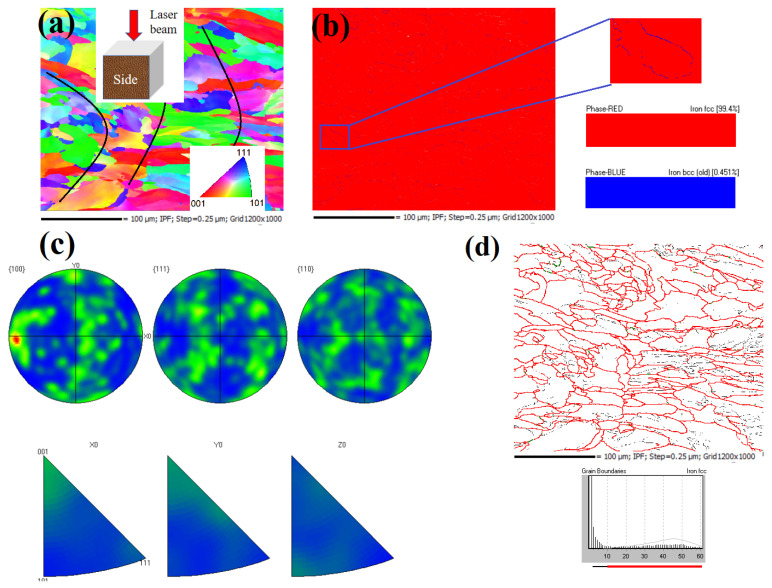
EBSD pattern: (**a**) orientation, (**b**) phase distribution, (**c**) pole and inverse pole, and (**d**) grain boundary of the SLM samples.

Figure 7a shows the grain alignment in the side of the as-built sample from the backscattered electron (BSE) signal, although the edge of the molten pool was blurred, and the fine grain was randomly aligned at the edge. The sizeable columnar crystal in the molten pool had a specific preferred orientation of <001>, which could also be confirmed from the pole figure and the reverse pole figure of the electron backscatter diffraction (EBSD) in Figure 7c. Moreover, the EBSD phase distribution in Figure 7b exhibited that 99.4% of the phase structure of the sample was FCC (austenite), and 0.45% of the phase structure was BCC (δ ferrite) [13,14], which were uniformly distributed along the austenite boundary(as shown in Figure 7d). The formed δ ferrite surrounded the austenite boundary and did not have enough time for a complete phase transition due to the extremely fast solidification rate. The solidification of austenite SS was not directly through the liquid eutectic reaction to form δ ferrite, but through the eutectic transition to form δ ferrite [22,23]. Given the relatively slower cooling rate, 304 L SS has sufficient time to transition from δ ferrite to γ austenite for conventional melting processes [22,23,24,25,26]. Under the condition of rapid cooling, the δ ferrites were skeleton-like, slate-like and block-like. With the increase of cooling rate, restricted diffusion leads to incomplete δ to γ transition, forming strip or even massive ferrite at an ultra-high super-cooling rate [22,25]. This phenomenon was also observed in the SLM process with a high cooling rate by Hou et al. [13]. 

**Figure 8:**
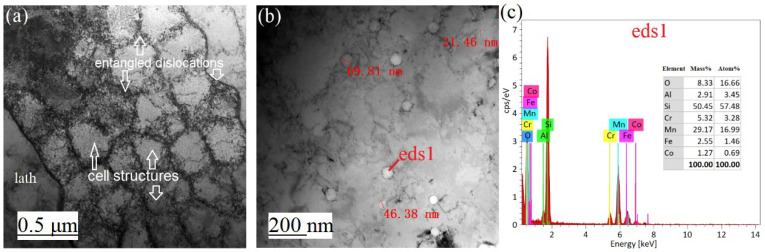
TEM analysis of SLM sample: (**a**) cellular structures and dislocations, (**b**) spherical nano-precipitates, (**c**) EDS of spherical nano-precipitate.

The detailed TEM morphology in Figure 8 exhibited that abundant dislocations existed between the laths and the cell structures, and a few entangled dislocations forming dislocation clusters. Nanoparticles were precipitated near the cell structure after rapid cooling, and the diameter of spherical particles ranged from 20 to 70 nm. The energy-dispersive spectroscopy (EDS) spectra verified that these particles were primarily composed of Mn, Si, and O. As the Si and Mn were oxidized by the remaining O_2_ in the SLM cavity filled with argon gas, Si-Mn-O rich nanoparticles were produced. Ghayoor et al. [12,14,15] found that the nanoparticle size was related to the oxygen content, and the nanoparticle size decreased when the residual oxygen in the SLM chamber was reduced. Therefore, finer nanoparticles were produced as the oxide residues are determined by the oxygen content, and can be eliminated by removing oxygen from the environment. 

### 3.5. Tensile Test and Fracture Analysis

**Figure 9:**
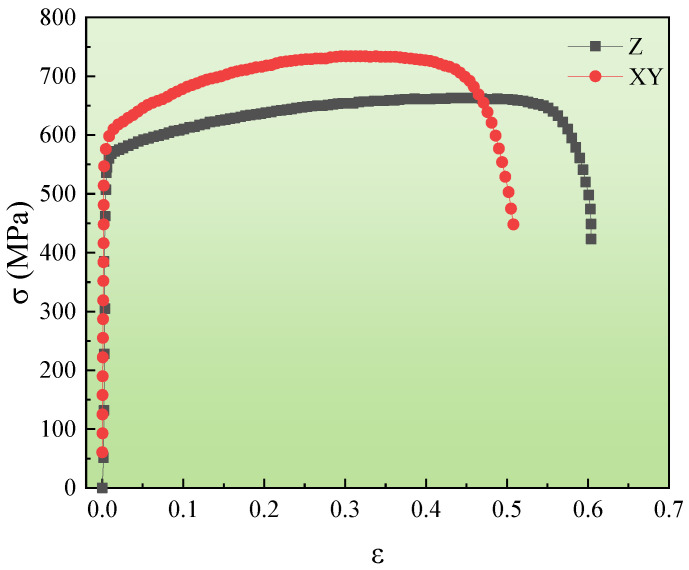
Engineering stress-strain curve of the specimen.

Samples produced under the optimal SLM condition were subjected to a tensile test (GB/T228.1-2010, China) with the tensile direction perpendicular (XY-sample) or parallel to the scanning direction (Z-sample). The stress-strain curves are presented in Figure 9. The elongation of the XY-sample was about 50%, which is less than that of the Z-sample of about 60%. However, its yield strength (595 MPa) and tensile strength (748 MPa) were much higher than the Z-specimen (yield strength of 573 MPa, tensile strength of 668 MPa). Therefore, the mechanical properties of the specimen were significantly anisotropic, depending on the printing directions, which was consistent with the results by DebRoy [20]. It is well known that solid crystal structures normally have anisotropic mechanical properties due to the anisotropic unit cell and defect distributions. Heat flows from the top to the bottom layer during additive manufacturing, creating a high thermal gradient following the build direction [27]. Austenite grains preferentially grow with the crystallographic orientation along the highest thermal gradient. Thus, a directional fibrous texture can be formed graphically in austenite along the manufacturing direction. Such a strong texture can lead to significant anisotropy in mechanical properties with the maximum difference between the parallel and perpendicular orientations in the AM parts. Furthermore, the non-uniform structure of metal parts can also lead to variations in tensile performance between fabricated parts with different build orientations, leading to anisotropic tensile properties.

**Figure 10:**
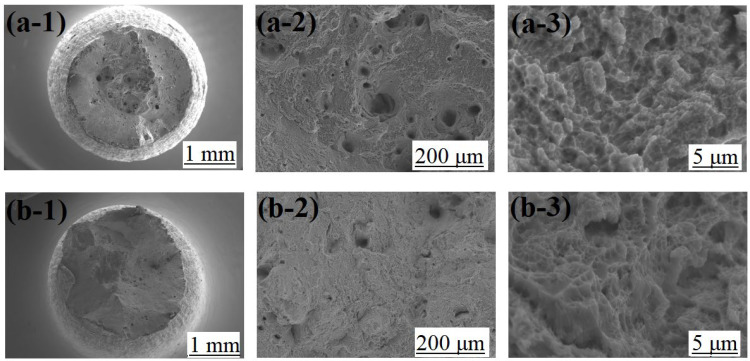
Fracture morphologies of the samples: (**a-1**) macroscopic fracture morphology of the Z-sample, (**a-2**) large dimples and holes of the Z-sample, (**a-3**) small dimples of the Z-sample; (**b-1**) macroscopic fracture morphology of the XY-sample, (**b-2**) large dimples and holes of the XY-sample, (**b-3**) small dimples of the XY-sample.

The fracture morphologies after tensile testing are shown in Figure 10. Necking and many large dimples with some holes in the fracture were observed in both XY and Z samples, as shown in Figure 10(a-1,b-1). Hence, the sample has undergone significant plastic deformation to form ductile fractures during the tensile testing. The magnified images in Figure 10(a-2,b-2) demonstrate that equi-axis large dimples, holes, and obvious tearing features emerged in the fibrous area. Among them, the number of large dimples and holes of the Z sample in Figure 10(a-2) was significantly larger than that of the XY sample in Figure 10(b-2). In addition, small pits were observed in the large holes for both samples, as shown in the further magnified SEM images in Figure 10(a-3,b-3). Affected by factors such as powder defects, forming environment and other parameters, various defects, such as spheroidization and pores, may be produced in additive manufacturing. Under external loading, micropores can be quickly created at these defects resulting in ultimate fracture with reduced tensile strength.

## 4. Conclusions

This study focused on the influences of different SLM parameters, including laser power, hatch space, and scanning speed, on the SLM 304L SS forming quality. A single-factor experiment and orthogonal analysis measure the density and hardness of the formed parts. The optimal SLM condition of 304L SS is identified. The microstructures and mechanical behavior of the SLM samples are characterized. Based on the experimental results, the following conclusions can be drawn:

(1) With the layer thickness of 0.03 mm, the relative optimal forming conditions require the heating power of 190 W, hatch space of 0.09 mm, and scanning speed of 800 mm/s. Under such conditions, the obtained RD was 99.24% and the hardness was 75 HRB, which was better than the other samples.
(2) Uniform and dense microcellular structures with larger columnar crystals are observed from a etched sample. While the fine grains are randomly oriented at the edge of the molten pool, the columnar crystals in the molten pool are aligned along the <001> direction. Many dislocations between the laths and the cell-like structures are entangled to form dislocation clusters. Spherical oxide nano-precipitates are formed near the cell-like structures.
(3) The highly anisotropic mechanical properties were determined by the manufacturing direction. The XY-sample has an elongation of 50%, which was significantly lower than the 60% of the Z-sample. The yield and tensile strength of the XY sample were 595 MPa and 748 MPa, respectively, which are higher than that of the Z-sample (the yield and tensile strength were 573 MPa and 668 MPa, respectively).

## Figures and Tables

**Table 1:** Elemental components of 304L SS powder (wt.%).

Cr	Ni	Mn	P	S	Si	O	C	Fe
19.15	9.54	1.04	0.009	0.004	0.38	0.066	0.01	Bal.

**Table 2:** The design and results of orthogonal experimental.

Number	Laser Power (w)	Hatch Space (mm)	Scanning Speed (mm/s)	RD (%)	Hardness (HRB)
1	1 (200)	1 (0.06)	1 (1000)	97.85624	69
2	1	2 (0.09)	2 (800)	99.06683	70
3	1	3 (0.12)	3 (600)	97.59142	71
4	2 (190)	1	2	99.04161	72
5	2	2	3	99.24338	75
6	2	3	1	98.42371	72.5
7	3 (170)	1	3	97.38966	68
8	3	2	1	97.61665	73
9	3	3	2	98.32282	71

**Table 3:** The range analysis of the orthogonal experiment.

	Actor	RD (%)			Hardness (HRB)		
Project		Laser Power	Hatch Space	Scanning Speed	Laser Power	Hatch Space	Scanning Speed
Mean 1		98.171	98.096	97.966	70	69.667	71.5
Mean 2		98.903	98.642	98.81	73.167	72.667	71
Mean 3		97.776	98.113	98.075	70.667	71.5	71.333
Range		1.127	0.546	0.844	3.167	3	0.5

## Data Availability

All data included in this study are available upon request by contact with the corresponding author.
